# RNAStructuromeDB: a transcriptome-wide database of predicted RNA secondary structures with integrated APIs for functional annotation and RNA-targeted drug discovery

**DOI:** 10.1093/nargab/lqag044

**Published:** 2026-04-30

**Authors:** Abdelraouf O Dapour, Jacopo Manigrasso, Warren B Rouse, Giuseppina La Sala, Miles V Aronnax, Leonardo De Maria, Russell S Hamilton, Sergio Martinez Cuesta, Anders Hogner, Walter N Moss

**Affiliations:** Bioinformatics and Computational Biology Program, Iowa State University, Ames, IA 50011, United States; Medicinal Chemistry, Research and Early Development, Cardiovascular, Renal and Metabolism (CVRM), BioPharmaceuticals R&D, AstraZeneca, Mölndal 431 50, Sweden; Roy J. Carver Department of Biochemistry, Biophysics and Molecular Biology, Iowa State University, Ames, IA 50011, United States; Medicinal Chemistry, Research and Early Development, Cardiovascular, Renal and Metabolism (CVRM), BioPharmaceuticals R&D, AstraZeneca, Mölndal 431 50, Sweden; Research IT, Iowa State University, Ames, IA 50011, United States; Medicinal Chemistry, Research and Development, Respiratory & Immunology (R&I), BioPharmaceuticals R&D, AstraZeneca, Mölndal 431 50, Sweden; Translational Science Experimental Medicine, Research and Development, Respiratory & Immunology (R&I), BioPharmaceuticals R&D, AstraZeneca, Cambridge 431 50, United Kingdom; Data Sciences and Quantitative Biology, Discovery Sciences, R&D BioPharmaceuticals, AstraZeneca, Cambridge CB2 0AA, United Kingdom; Medicinal Chemistry, Research and Early Development, Cardiovascular, Renal and Metabolism (CVRM), BioPharmaceuticals R&D, AstraZeneca, Mölndal 431 50, Sweden; Roy J. Carver Department of Biochemistry, Biophysics and Molecular Biology, Iowa State University, Ames, IA 50011, United States

## Abstract

RNA structure critically governs biological function in both physiological and pathological contexts, making high-resolution structural maps essential for RNA-targeted therapeutics. Yet, despite recent advances, well-validated structural targets for drug design remain limited. To help bridge this gap, we generated the first genome-scale map of the human RNA structurome by applying ScanFold to >230 000 annotated human pre-mRNA transcripts, identifying sequences likely evolved to form highly stable and functional secondary structures. We also performed a global analysis of regions with *z*-scores ≤ –2 and statistically characterized their two-dimensional folding patterns. In addition, we developed the RNA-Annotator Pipeline to integrate 20 diverse biological annotations, such as tissue-specific expression and protein interactions, with the structural data. Our results reveal local folding propensities and unusually stable structures with high-confidence architectures, providing insights for prioritizing RNA targets and guiding therapeutic design, including antisense oligonucleotides and small molecules. All ScanFold results are publicly available through RNAStructuromeDB. Using the RNA-Annotator Pipeline, analysis of SMN1 and SMN2 pre-mRNAs showed that a single C-to-T transition in SMN2 induces structural rearrangements that disrupt a critical splicing enhancer. This toolkit establishes an integrated workflow that enables researchers to explore RNA structure–function relationships and accelerate advances in RNA-targeted drug discovery and RNA biology.

## Graphical Abstract

**Graphical Abstract:**
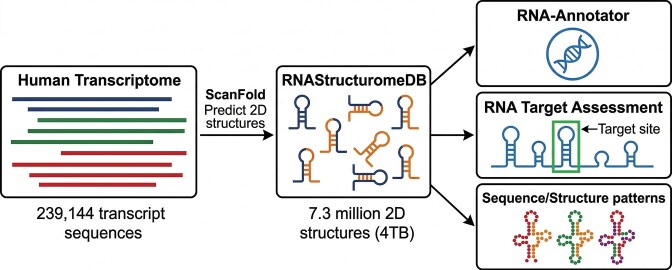

## Introduction

At some level RNA is a fundamental component of every biological process. Every role of RNA is affected by its structure, which can modulate stability, processing, expression, and localization through alteration of its intrinsic properties or its interactions with other molecules [1–3]. Thus, a comprehensive understanding of biology necessitates a better understanding of RNA structure. Unlike proteins, where every residue of a polypeptide sequence is (almost always) ordered to form a stable 3D structure with a given function [4], regions of defined structure and function are typically interspersed throughout large, predominantly unstructured, RNAs. Moreover, RNA is intrinsically more flexible than proteins, sometimes adopting multiple valid conformations for a single sequence [3, 5]. Furthermore, RNA structures in long transcripts can be greatly altered by co-transcriptional folding, post-transcriptional processing, intermolecular interactions, and refolding events, which can continue across a RNA’s lifetime [6–8].

For the reasons outlined above, the application of 3D prediction tools (like Alphafold3) to large RNAs is impractical [9]. The hierarchical nature of RNA folding, however, enables the extraction of useful information from its secondary (2D) structure. The 2D structure of RNA provides the bulk of its thermodynamic stability and defines the specific arrangement of residues of the transcript, which in-turn, largely defines its allowable 3D conformation. Thus, useful biological information can be derived from RNA 2D structure, allowing the formulation of various biological hypotheses. Various algorithms have been developed to predict RNA 2D structure, where most rely on the calculation of the Gibbs folding free energy change (Δ*G*°) of going from an unstructured to a fully folded RNA. A widely applied approach is to predict the minimum free energy (MFE) RNA 2D structure: the conformation that has the lowest (most favorable) Δ*G*° at thermodynamic equilibrium, which is taken to be the most likely native 2D structure of the RNA [10].

While the MFE approach has enabled numerous important scientific discoveries, limitations in accuracy typically necessitate the application of various supplementary techniques to improve 2D predictions [11–16]. The identification of functional RNA structures with strict conformational requirements remains an important challenge. Even when a candidate RNA is identified, its sheer length (often thousands of nucleotides) presents an immense hurdle: systematically mapping the critical functional domains by experimental testing is infeasible [17]. Focusing structural analysis therefore requires a strategy to prioritize high-value regions for targeted therapeutic strategies, including small molecules [18–20] and antisense oligonucleotides (ASOs) [21, 22].

To address these needs, we previously developed the ScanFold program [23, 24] to fold and find highly ordered local RNA structures that have likely evolved for structure/function. In this approach, a large RNA is analyzed using a sliding window approach, where for each window an MFE structure is predicted alongside other metrics derived from the thermodynamic analysis. The key metric here is the *z*-score, which measures the sequence-ordered (evolved) thermodynamic stability bias of RNA, which is used to identify and model short local 2D structures that are likely to fold in the cell. ScanFold has been successfully applied toward structured RNA discovery in human genes [25–29] and the genomes of various human pathogens [23, 30–32].

In this current study, we address the dual challenge of structural characterization and target prioritization by applying ScanFold to the entire human transcriptome. The resulting data are made available as a comprehensive resource in the RNAStructuromeDB. While an earlier version of the RNAStructuromeDB [33] analyzed a limited set of mature human RNAs, the present work expands it to the entire human pre-mRNA transcriptome, capturing structures across introns, exons, and UTRs for a more complete view of RNA folding, thus providing a genome-scale map of predicted RNA structures. To navigate the massive RNAStructuromeDB dataset, and enable the filtering critical to target discovery, we developed an analytical toolkit for the structural and functional annotation of any transcript or region of interest. Specifically, the RNA-Annotator pipeline is provided to facilitate functional annotation and cross-referencing of ScanFold structural data with complementary “omics” data.

RNA-Annotator integrates 20 high-confidence annotation layers, including RBP occupancy (eCLIP), disease-associated variants (ClinVar), tissue-specific expression (GTEx), alternative splicing events, chemical modifications, evolutionary conservation, *in vivo* probing reactivity (DMS/SHAPE), enzymatic probing tracks such as tNET RNase-seq, an RNase-based cleavage/accessibility profiling assay, retrieved via RASP v2.0 [34], and many others, directly onto user-defined transcripts or genomic intervals. This automated overlay generates a richly annotated structural landscape, revealing, for example, stable hairpins that overlap exonic splicing enhancers, stems harboring pathogenic variants, or RBP-bound motifs critical for isoform regulation. By co-locating structural predictions with experimentally derived functional signals, RNA-Annotator enables investigators to evaluate candidate motifs against established criteria for druggability—stability, accessibility, conservation, and regulatory impact—without imposing predefined filtering thresholds. Thus, while ScanFold establishes the structural foundation, RNA-Annotator delivers comprehensive, multilayered annotation, empowering precise, and evidence-driven selection of RNA targets for small-molecule or ASO development. To demonstrate the platform’s utility, we analyzed the well-studied SMN1 and SMN2 pre-mRNAs, showing how the RNA-Annotator pipeline seamlessly pinpoints the single-nucleotide C-to-T transition in *SMN2* that induces structural rearrangements and disrupts a critical splicing enhancer, consistent with established findings.

Taken together, the RNAStructuromeDB and these complementary annotation and analysis toolkits, offers a comprehensive pipeline to streamline the discovery of functionally important and potentially druggable RNA structures, moving from the entire transcriptome down to specific, high-value targets with speed and precision.

## Materials and methods

### Data sources

Human transcriptome data were sourced from the GENCODE database (release 45; Accession GRCh38.p14; [35]) on January 23, 2024. A single fasta file containing the sequences of all pre-mRNA isoforms was downloaded, split into individual fasta files, and used as input for ScanFold. We implemented filtering criteria to ensure computational feasibility, excluding transcripts shorter than 120 nucleotides or longer than 200 000 nucleotides from downstream analysis. A limitation in the sub-motif extraction script, related to parenthesis parsing for certain complex structures, was noted. This arises because ScanFold assigns all possible pairing partners for each nucleotide while calculating Zsum and Znorm values. In rare cases, a nucleotide may be assigned the best (lowest) Znorm score with more than one potential partner—an arrangement that is not biologically valid. In the current version of ScanFold, such instances are handled by ignoring nucleotides with multiple competing pairing partners, thereby leaving them unpaired.

Comprehensive eCLIP datasets for 169 RNA-binding proteins (RBPs), comprising 250 experiments in K562 and HepG2 cells, were acquired from the ENCODE project portal [36]. To create a high-confidence dataset, we applied three key filters: first, we included only experiments mapped to the GRCh38 human genome assembly. Second, to ensure reproducibility, we retained only peaks that passed ENCODE’s Irreproducible Discovery Rate (IDR) analysis, which identifies binding sites consistently detected in at least two biological replicates. Third, we used ENCODE’s IDR-filtered narrowPeak files, which capture discrete RBP-binding sites.

Functional annotations were retrieved from the NCBI RefSeq Functional Elements collection (Annotation Release 110, GRCh38), which includes four primary data types: biological regions (e.g. promoters), discrete functional features, recombination partners, and long-range regulatory interactions [37].

Additional datasets were sourced from established public databases. Experimentally validated microRNA (miRNA) annotations were obtained from miRBase (release 22.1) [38] and predicted miRNA binding sites were from TargetScanHuman (release 8.0) [39]. Data for RNA modifications were downloaded from RMBase v2.0 and consolidated into a single curated file [40]. Chemical probing data from 178 distinct studies were sourced from the RASP v2.0 database [34], and polyadenylation sites were acquired from the PolyASite 2.0 atlas [41]. To incorporate clinically relevant variants, we obtained data from NCBI’s ClinVar database [42]. Evolutionary conservation scores were sourced from the UCSC phastCons track (100-way vertebrate alignment) [43, 44].

Additional layers of regulatory and functional context were integrated from the UCSC Genome Browser database [45]. To provide insight into genomic features, such as transcriptional regulation, we included annotations for CpG islands and experimentally verified transcription factor binding sites (TFBS). Information on RNA processing and isoform diversity was incorporated by extracting data on known splice variants from SpliceVarDB and alternative splicing events from the Alt Events track. Finally, to enable tissue-specific analyses, we integrated RNA expression data from the Genotype-Tissue Expression (GTEx) project.

For annotations that are frequently updated, our pipeline directly integrates the public REST APIs from Dfam [46] and Ensembl [47] to retrieve repetitive elements and single nucleotide polymorphisms (SNPs) on-the-fly.

A key feature of the RNA-Annotator pipeline is its direct integration within the updated RNAStructuromeDB. To facilitate seamless structural analysis, the tool automates the entire workflow for any user-provided transcript ID. Upon execution with the -download_scanfold flag, the pipeline queries the database, downloads the corresponding pre-computed ScanFold data package, and unzips the contents. Subsequently, a suite of dedicated flags (-scanfold_bp, -scanfold_mfe, -scanfold_zscore, -scanfold_ed) triggers the automated processing of these raw outputs into IGV-ready visualization tracks. This includes converting the ScanFold base-pairing models into genomic coordinates for arc visualization, and formatting the per-nucleotide MFE, *z*-score, and Ensemble Diversity files into correctly anchored WIG tracks. This integration transforms the static database into a dynamic resource, allowing researchers to rapidly generate and overlay deep structural information onto a rich canvas of functional annotations for any transcript of interest.

All static datasets originally provided on the hg19/GRCh37 assembly were updated to GRCh38 using the UCSC LiftOver tool [48]. Source files in indexed binary formats (e.g. bigWig and bigBed) were converted to standard text-based formats (. bedGraph,. bed) using UCSC utilities [49]. To facilitate fast, local querying by our API, all large, compressed data files are provided with their corresponding tabix index (.tbi) files. The complete, final curated collection of all datasets used in this study is available for download as a single compressed folder (resources_data_sets) from Zenodo (https://doi.org/10.5281/zenodo.16953760).

### Computational RNA secondary structure prediction with Scanfold

To identify stable and potentially functional RNA secondary structures within the human transcriptome, we utilized the ScanFold 2.0 pipeline [24]. ScanFold operates in two sequential phases: a scanning phase and a subsequent folding and consensus building phase. In the initial ScanFold-Scan phase, a sliding window is applied across the entire RNA sequence. For each window, the MFE and base-pairing scheme are computed using RNAfold 2.0 [50], implementing the Turner thermodynamic model [10]. To assess the intrinsic stability bias of the native sequence, a set of randomized sequences (generated via mononucleotide or dinucleotide shuffling) are also folded, and their average MFE is determined. This allows for the calculation of a thermodynamic *z*-score for each window.

The subsequent ScanFold-Fold phase processes these *z*-scores to derive consensus secondary structures. This is achieved by identifying base pairs that are consistently predicted across multiple low *z*-score windows. A critical aspect of this step is the refinement of pairing partners using the Znorm metric. Znorm normalizes the Zsum (which aggregates all predicted base-pairing occurrences for a given nucleotide) by the number of windows in which that nucleotide participates in a pair. This normalization is crucial because it provides a coverage-adjusted *z*-score, enabling a more accurate comparison of structural elements regardless of their position within the transcript (e.g. segments near the ends of the transcript, which have fewer overlapping windows). The base pair arrangement yielding the lowest Znorm is selected as the most favored, forming the basis of the final *z*-score weighted consensus model. These resulting ScanFold structures are inherently biased towards sequences exhibiting ordered stability, a characteristic often indicative of biological function. We extracted all predicted structures containing at least one base pair with a *z*-score of ≤−2 for downstream analysis.

ScanFold provides several key structural metrics. The MFE (or Δ*G)* quantifies the thermodynamic stability of the predicted structure, with more negative values signifying greater stability. The *z*-score is a normalized measure of ordered stability, where negative values indicate how many standard deviations more stable the native sequence is compared to random sequences of the same nucleotide composition, thereby suggesting potential functional significance. Ensemble diversity (ED), derived from the RNA partition function, quantifies the conformational landscape of a predicted structure where lower values point to a single dominant conformation and higher values imply a more flexible or poorly defined structure. Arc diagrams are employed to visualize the weighted z-score structures, with distinct color coding: blue arcs denote *z*-scores ≤−2, green arcs represent *z*-scores between −1 and −1.99, and yellow arcs indicate *z*-scores between 0 and −0.99. For further details on the ScanFold algorithm, its output formats, and their interpretation, refer to the original publications [23, 24]. In this current analysis we used a 120-nucleotide scanning window length and 1-nucleotide step size, consistent with previous benchmarking showing that this window size robustly captures local structural signals while limiting spurious long-range pairings [51]. Previous analyses showed that ∼60–150 nt windows tend to best agree with probing-informed structures [23, 30, 52]. While long-range base pairing can play important functional roles, a significant fraction of functional RNA structures in long, co-transcriptionally folding pre-mRNAs are local; therefore, ScanFold data provide transcriptome-wide maps of local stability/structure to highlight these local regions of high interest.

### RNAStructuromeDB database construction

The ScanFold analysis of the entire human transcriptome was automated and executed on a high-performance computing (HPC) cluster. To optimize resource allocation and job scheduling, transcripts were partitioned into batches based on their sequence length (small, medium, and large). Small and medium transcript batches (200 sequences <20 000-nucleotide and 50 sequences from 20 001–199 999-nucleotide, respectively) were processed in highly parallelized jobs, utilizing approximately 100 CPUs per job, with typical run times of 3–5 days. In contrast, the largest transcripts batches (10 sequence >200 000-nucleotide) required dedicated, longer-running jobs, sometimes requiring >30 days on a similar number of cores. The full transcriptome analysis was completed over approximately two months. A monitoring system was used to identify and automatically resubmit jobs that failed due to transient server or node issues, ensuring the comprehensive processing of all transcripts.

### “Dictionary” of RNA substructures

To create a comprehensive dictionary of RNA secondary structure motifs, our analysis was restricted to the MANE (Matched Annotation from NCBI and EBI) human transcriptome. Although ScanFold folding was performed transcriptome-wide on the full GENCODE set (∼230 000 pre-mRNA isoforms), we restricted this “Dictionary” analysis to MANE to use one representative transcript per gene, avoiding redundancy and bias from genes with many isoforms. We first applied a custom script (Supplementary File S1) to parse the dot-bracket notation of all ScanFold-predicted structures. This script identifies discrete base-paired motifs (e.g. hairpins, stems) by incrementing a bond order counter for each opening parenthesis “(” and decrementing for each closing “)”. A motif is defined as the sequence and structure between the point where the bond order becomes greater than zero and subsequently returns to zero, with an optional flanking region (default = 0 nucleotides) to preserve sequence context. This global extraction was performed on structures filtered at two *z*-score thresholds: ≤ −1 and a more stringent ≤ −2.

Following the global extraction, we mapped the genomic locations of these sub-motifs. Genomic annotations were obtained from the MANE.GRCh38.v1.3.ensembl_genomic.gff.gz file (downloaded on March 9, 2024). This file was processed using gffutils (v0.13) and a custom script (Supplementary File S2) to extract the precise coordinates for all introns, exons, 5′ UTRs, and 3′ UTRs of MANE transcripts. Subsequently, a series of scripts (Supplementary Files S3–S6) were used to assign each previously identified sub-motif to its corresponding transcript region. For each sub-motif, we cataloged its sequence, dot-bracket structure, MFE, ScanFold-derived constraints, and ensemble diversity into comprehensive supplementary output files, generated for both the −1 and −2 *z*-score thresholds to facilitate deeper analyses.

### Analysis of global structural features

Folded regions with *z*-scores below −2, as determined by ScanFold, were compiled into a dataset of over 2.5 million sequence-structure pairs from the 3′- and 5′-UTRs, as well as introns and exons. A *z*-score cutoff of −2 or less is used as a practical “rule-of-thumb” filter to prioritize the most unusually stable (i.e. highly structured) pairs. While *z*-scores ≤ −1 can also be informative for broader structural discovery, they are considered secondary candidates. We developed a custom Python pipeline using modified functions from the IPyRSSA library [53] to analyze this dataset.

The pipeline systematically extracts and characterizes RNA secondary structure elements from sequence-structure pairs, focusing on three key components: multibranch loops, their basal stems, and hairpins, along with their associated substructural motifs. Briefly, stems and hairpins are classified into two categories: those that are fully base-paired and those containing substructures such as bulges or internal loops. For hairpin analysis, the pipeline examines the properties of apical loops, including length and sequence composition. When hairpins contain substructures, the algorithm calculates spatial distances to identify which feature lies closest to the apical loop.

Structural elements are organized by type and length into distinct categories, with data presented as percentages of total structures in each transcript region. Sequence conservation within each structural category is evaluated through LOGO analysis [54] to reveal functional sequence-structure constraints and evolutionary patterns. Results focus on 5′ UTR data unless otherwise noted, given the consistent patterns observed across datasets. The Python pipeline is available for download from GitHub.

### The RNA-Annotator pipeline

To facilitate the integrated analysis of the curated datasets, we developed RNA-Annotator, a command-line tool designed to automate the aggregation, processing, and visualization of functional and genomic annotations. The tool is implemented in Python 3 and leverages standard scientific libraries, including Pandas for data manipulation and Requests for API communication. It is designed for full cross-platform compatibility with Windows, macOS, and Linux operating systems. The pipeline is executed from the command line and accepts as primary input either genomic coordinates (e.g. chr1:1000–2000) or an Ensembl Transcript ID (e.g. ENST00000263100). If a transcript ID is provided, the tool performs a look up against a local mapping file derived from Ensembl BioMart to resolve the corresponding genomic coordinates. For querying local, indexed data files, the pipeline utilizes the command-line tools bedtools and tabix via Python’s subprocess module. To ensure cross-platform compatibility and avoid reliance on shell-specific features like process substitution, the query region is written to a secure temporary file which is then passed as an argument to the external tool. This design allows the core analysis functions to operate identically across all major operating systems. API-based queries for real-time data retrieval are handled using the Python requests library.

Upon execution, RNA-Annotator generates two output subdirectories. The results/detailed_results/ folder contains raw data in tabular formats (.tsv, .csv) for downstream analysis, while the results/bed_tracks/ folder contains files formatted for direct visualization as tracks in a genome browser, including .bed, .wig, and .bedGraph formats. The pipeline also includes an optional feature to automatically visualize these tracks in the Integrative Genomics Viewer (IGV). This is achieved by first launching the IGV application using a platform-aware method and subsequently communicating with the running instance via its command port (default 60151) to load all generated tracks. This remote-control functionality is handled by the netcat utility and is currently supported on macOS and Linux.

The complete RNA-Annotator source code, a Conda environment file for simple one-step installation of all dependencies, and a comprehensive user manual are freely available under the MIT License at our GitHub repository: https://github.com/moss-lab/RNA-Annotator-v1.

## Results

### Overview of the human RNA StructuromeDB

To create a comprehensive map of RNA secondary structures across the human transcriptome, we analyzed 239 144 transcript isoforms from the GENCODE database. After applying length-based filters, 230 061 transcripts were successfully processed through our ScanFold pipeline, generating full structural models and metrics. Of these, 228 217 transcripts yielded complete sub-motif extractions, while 1844 could not be processed for sub-motifs due to parsing limitations, though all other structural data were successfully generated. These 230 061 entries represent the full set of annotated GENCODE transcript isoforms (including alternative splicing/annotated alternative TSS isoforms) that passed filtering; we did not attempt to infer or model unannotated/novel TSS variants beyond the GENCODE annotation.

This complete dataset, comprising approximately 4 terabytes of data, has been made publicly available through the RNAStructuromeDB web portal at https://structurome.bb.iastate.edu/azt. The database features a search function allowing users to query by transcript ID. For each transcript, a downloadable ZIP archive is provided, containing seven key output files, including the full sequence (.fasta), windowed scan results (.tsv), extracted sub-motifs (.txt), and the final consensus structures in dot-bracket (.dbn) and CT (.ct) formats for both –1 and –2 *z*-score thresholds. In addition to this, a folder containing genome browser tracks (e.g. for IGV visualization) is also available. All other data, such as intermediate ScanFold outputs (including per-nucleotide pairing annotations), and structure predictions with no *z*-score filter, are stored in-house to maintain a streamlined user interface and are available upon request.

**Figure 1.**
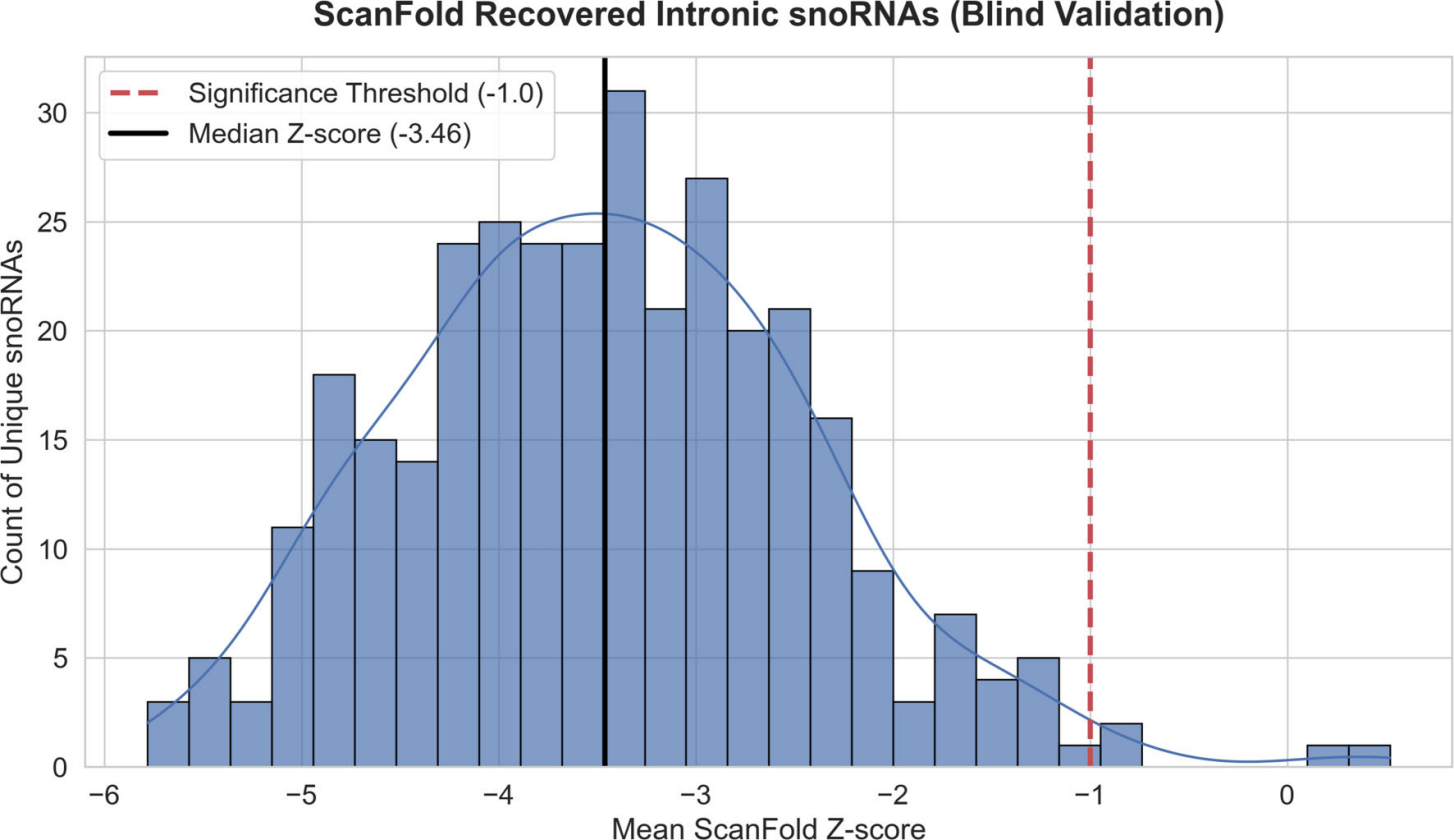
Blind recovery of intronic snoRNAs validates ScanFold structural predictions. Histogram displaying the distribution of mean thermodynamic *z*-scores for 335 unique snoRNAs recovered from the transcriptome-wide ScanFold analysis. These functional RNAs, located within pre-mRNA introns, were identified solely by intersecting ScanFold-predicted structural motifs with GENCODE annotations, without prior training or specific targeting. The red dashed line marks the *z*-score significance threshold of –1.0, while the black solid line indicates the population median of –3.46. The strong skew toward highly negative *z*-scores (with 99% of recovered motifs scoring ≤ –1.0) demonstrates the pipeline’s capability to accurately distinguish stable, evolved structural elements from the surrounding intronic background.

To validate the pipeline’s ability to distinguish evolved, functional structures from the surrounding intronic background, we performed a blind intersection of our predicted structural motifs against annotated small nucleolar RNAs (snoRNAs). Because our dataset comprises full pre-mRNAs, these snoRNAs represent embedded “structural islands” hidden within massive intronic regions. Our analysis successfully recovered 335 unique snoRNAs that were consistently identified as distinct structural motifs across multiple transcript isoforms. Strikingly, these blindly recovered regions exhibited exceptional thermodynamic stability, with a median *z*-score of –3.46 (Fig. 1). Furthermore, 93% (311/335) of these motifs possessed a *z*-score ≤ –2.0, and 99% scored ≤ –1.0. These results demonstrate that ScanFold effectively decouples functional structural signal from intronic noise, consistently identifying stable molecular architectures regardless of the flanking isoform-specific sequence context.

### A dictionary of 7.3 million RNA structures reveals a biased distribution across transcript regions

Applying our structural parsing pipeline to ScanFold-predicted structures across the MANE transcriptome identified a total of 7 344 550 structures using a *z*-score threshold of ≤ −1 (Supplementary File S7). Applying a more stringent *z*-score filter of ≤ −2 yielded a subset of 2 639 616 highly stable structures (Supplementary File S8).

Annotation of the genomic coordinates for the −1 *z*-score sub-motifs revealed a distinct distribution across transcript regions. A total of 2 598 039 sub-motifs were located in 3′UTRs and 2 348 155 in introns, highlighting the structural complexity of these regions. In contrast, only 70 236 were found in exons and a mere 7371 in 5′UTRs. The low count of sub-motifs in 5′UTRs is consistent with the biological constraint against stable structures in these regions that could hinder ribosome binding and translation initiation, suggesting a functional investigation is warranted for those that are present. Motifs that spanned multiple genomic regions were not assigned to a single category but were included in the comprehensive supplementary data files available for deeper analysis.

### Global characteristics of the human RNA structurome

**Figure 2.**
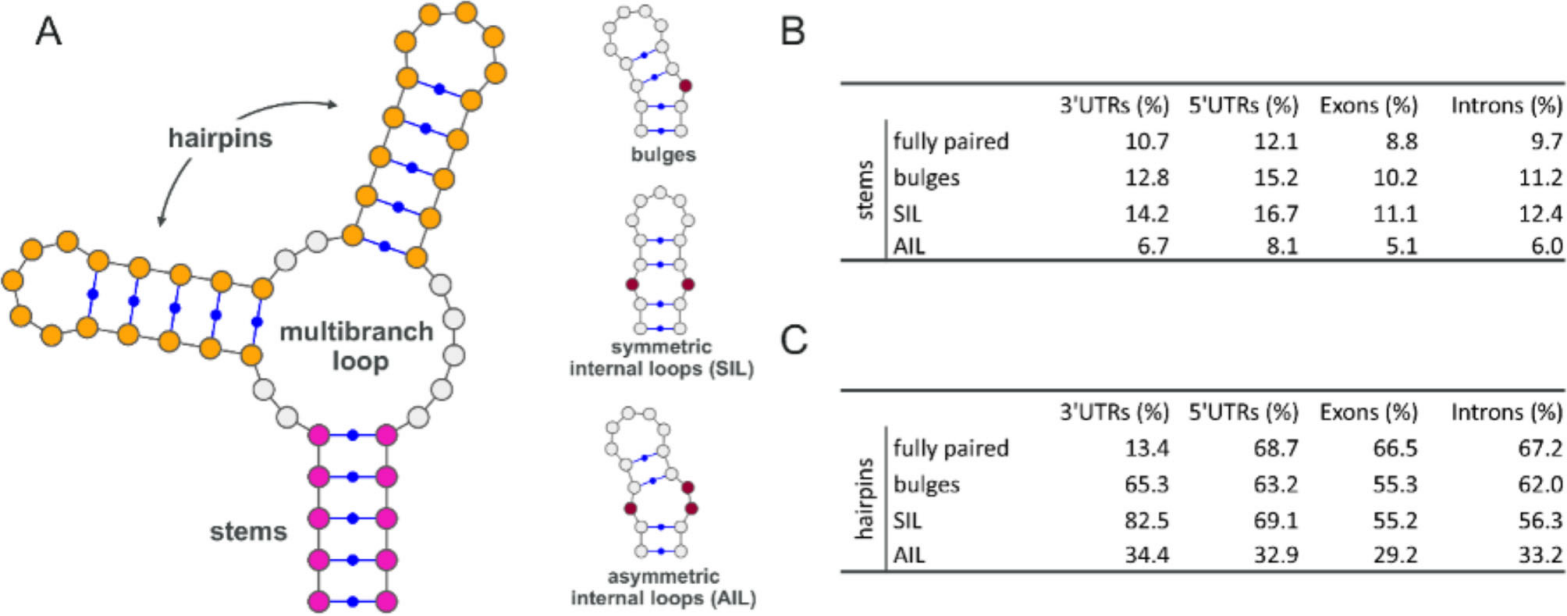
Overview of RNA secondary structure analysis. (**A**) Classification of characterized structural motifs: hairpins, stems, multi-branch loops, bulges, symmetric internal loops (SIL), and asymmetric internal loops (AIL). (**B** and **C**) Distribution of stems, hairpins, and their substructures across different transcript regions. Data represent percentages of total structures analyzed in each transcript region.

To illustrate the insights achievable with our RNAStructuromeDB for transcriptome-wide analysis, we analyzed over 2.5 million RNA secondary structures and associated sequence, focusing on stems that close multibranch loops, hairpins, and their substructural elements (motifs), including bulges, internal loops, and hairpin loops, thereby revealing key features of human transcriptome structural organization (Fig. 2A).

We observed distinct patterns in the abundance of RNA structures across different transcript regions. Multibranch loops showed the highest prevalence in 5′UTRs (52.1%), followed by 3′UTRs (44.3%), introns (39.3%), and exons (35.2%), consistent with the regulatory roles of complex secondary structures in untranslated regions. Within these multibranch structures, fully base-paired stems, bulges, symmetric internal loops, and asymmetric internal loops were differentially distributed (Fig. 2B). The 5′UTRs demonstrated enrichment in all structural motifs compared to other transcript regions (Fig. 2B). Fully base-paired stems comprised 12.1% of structures in 5′UTRs versus 10.7% in 3′UTRs, 9.7% in introns, and 8.8% in exons. Bulges were most abundant in 5′UTRs (15.2%) compared to 3′UTRs (12.8%), introns (11.2%), and exons (10.2%). Symmetric internal loops followed a similar pattern with 16.7% prevalence in 5′UTRs versus 14.2%, 11.1%, and 12.4% in 3′UTRs, exons, and introns, respectively. Asymmetric internal loops were consistently the least abundant structural motif across all transcript regions, ranging from 8.1% in 5′UTRs to 5.1% in exons.

**Figure 3.**
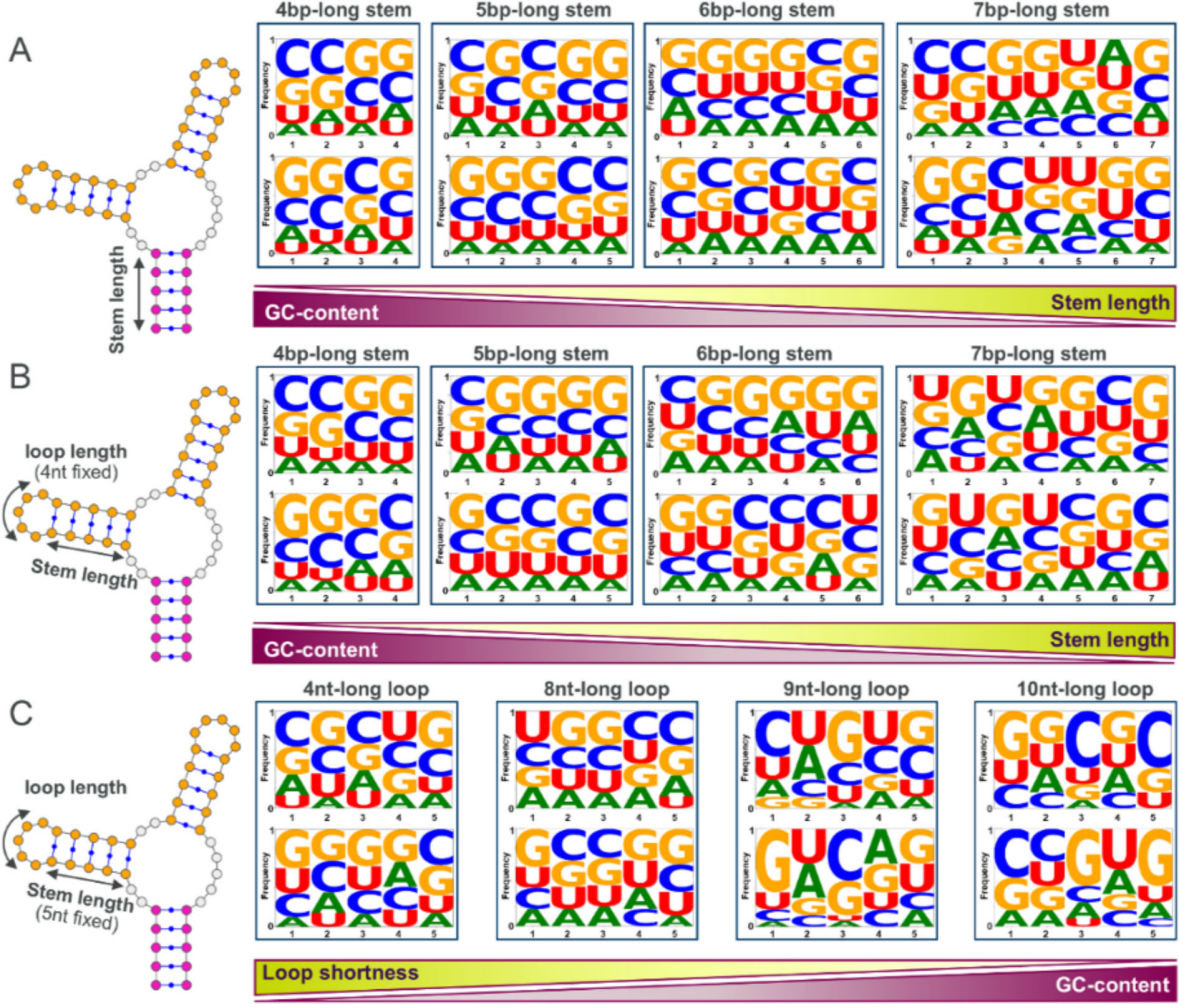
Sequence–structure constraints and patterns in the human structureome. (**A**) LOGO analysis of 4-to-7 base pair-long stems closing multibranch loops showing the inverse relationship between stem length and GC content. Shorter stems require higher GC content for stability. Top and bottom panels show complementary 3′ and 5′ sequences, respectively. (**B**) LOGO analysis of 4-to-7 base pair-long hairpin stems with apical tetraloops, demonstrating decreased GC content with increasing stem length, consistent with panel (A). (**C**) LOGO analysis of 5 base pair-long hairpin stems across hairpins with 4-to-10 nucleotide-long apical loops. GC content increases with loop length, indicating that larger apical loops require enhanced stem stabilization.

Fully base-paired stems closing multibranch loops predominantly spanned 4–9 base pairs (Supplementary Fig. S1A). While stems in 3′UTRs and introns could extend up to 46 nucleotides in length, such extended structures were rare. These long stems may represent fragments of repeat sequences, such as transposons or other inverted repeats, which are capable of forming extended base-paired structures; however, such instances remain rare with only four instances of 46-nucleotide stems identified in intronic transcript regions. Notably, further sequence–structure analysis revealed an inverse relationship between stem length and GC content, with shorter stems displaying higher GC content, likely to compensate for reduced length and maintain structural stability for proper folding (Fig. 3A).

Analysis of bulge distribution revealed that single-nucleotide bulges are prevalent, with a sharp decline in abundance as bulge length increased (Supplementary Fig. S1B). Sequence composition analysis showed that 1-nucleotide bulges were predominantly formed by adenine (A) or uracil (U) residues (Supplementary Fig. S2A). Two-nucleotide and longer bulges showed progressively lower abundance, consistent with the principle that larger disruptions in base pairing are energetically less favorable. Symmetric internal loops displayed a similar length-dependent abundance pattern, with shorter loops being more prevalent than longer ones (Supplementary Fig. S1C). Sequence analysis of 1 × 1 symmetric loops revealed notable enrichment of guanosine residues, comprising up to 40% of these structures (Supplementary Fig. S2B). Asymmetric internal loops consistently represented the least abundant structural motif class, suggesting either reduced functional importance or increased evolutionary pressure against such asymmetric disruptions.

Hairpins, whether occurring as standalone structures or as components of multibranch loops, exhibited markedly different distribution patterns compared to stems closing multibranch loops, with hairpins being significantly more abundant than stems that close multibranch loops (Fig. 2C). The 3′UTRs showed distinct depletion in fully base-paired (lacking internal loops/bulges) hairpins (13.4%) compared to other transcript regions (68.7% in 5′UTRs, 66.5% in exons, and 67.2% in introns). Within hairpin structures, bulges, symmetric internal loops, and asymmetric internal loops were significantly more abundant than in stem structures. Symmetric internal loops reached 82.5% abundance in 3′UTR hairpins compared to 69.1% in 5′UTRs, 56.3% in introns, and 55.2% in exons, possibly suggesting distinct functional roles for these substructural motifs in different transcript regions.

Importantly, we observed functional relationships between apical loop length, hairpin stem length, and GC content in fully base-paired hairpins, revealing two distinct compensatory mechanisms. First, hairpin stem GC content showed an inverse relationship with stem length (Fig. 3B): shorter stems compensate for their reduced base-pairing stability by incorporating more GC pairs, which form three hydrogen bonds compared to two in AU pairs. Second, stem GC content displayed a positive correlation with apical loop length (Fig. 3C): hairpins with larger apical loops require more stable, GC-rich stems to counterbalance the destabilizing effect of the extended single-stranded loop region. These relationships indicate that RNA structures maintain overall thermodynamic stability through local sequence adjustments, with stem composition adapting to compensate for variations in both stem and loop architecture, in line with the experimentally observed local stability compensation mechanism [55].

Nonetheless, we observed that hairpin loop length distribution favored shorter loops, with tetraloops being most common (Supplementary Fig. S3A). Single-nucleotide bulges and 1 × 1 symmetric internal loops were the most representative of their respective classes (Supplementary Figs S3B and C, and S4A and B), similar to findings for stems closing multibranch loops. Notably, the spatial organization of such structural motifs within hairpins revealed characteristic positioning patterns. Single-nucleotide bulges were typically found 2–3 base pairs away from apical loops, with a notable exception for triloops, which were separated by 7 base pairs from the next single-nucleotide bulge (Supplementary Fig. S5). Two-nucleotide bulges showed differential positioning depending on apical loop size: they were found 3–4 base pairs from apical loops of up to 6 nucleotides, but 5–7 base pairs from longer apical loops. Three-nucleotide bulges displayed inverse positioning preferences, being located 2–4 base pairs from large apical loops but 3–11 base pairs from shorter apical loops. Both symmetric and asymmetric internal loops within hairpins showed preferential positioning 2–3 base pairs from apical loops (Supplementary Fig. S6).

Taken together, these findings provide comprehensive insights into the organizational principles governing RNA secondary structure formation in the human transcriptome and suggest potential functional and therapeutic implications for different structural motifs across transcript regions.

### Case study: RNA-Annotator reveals the disease-causing structural switch in SMN2

**Figure 4.**
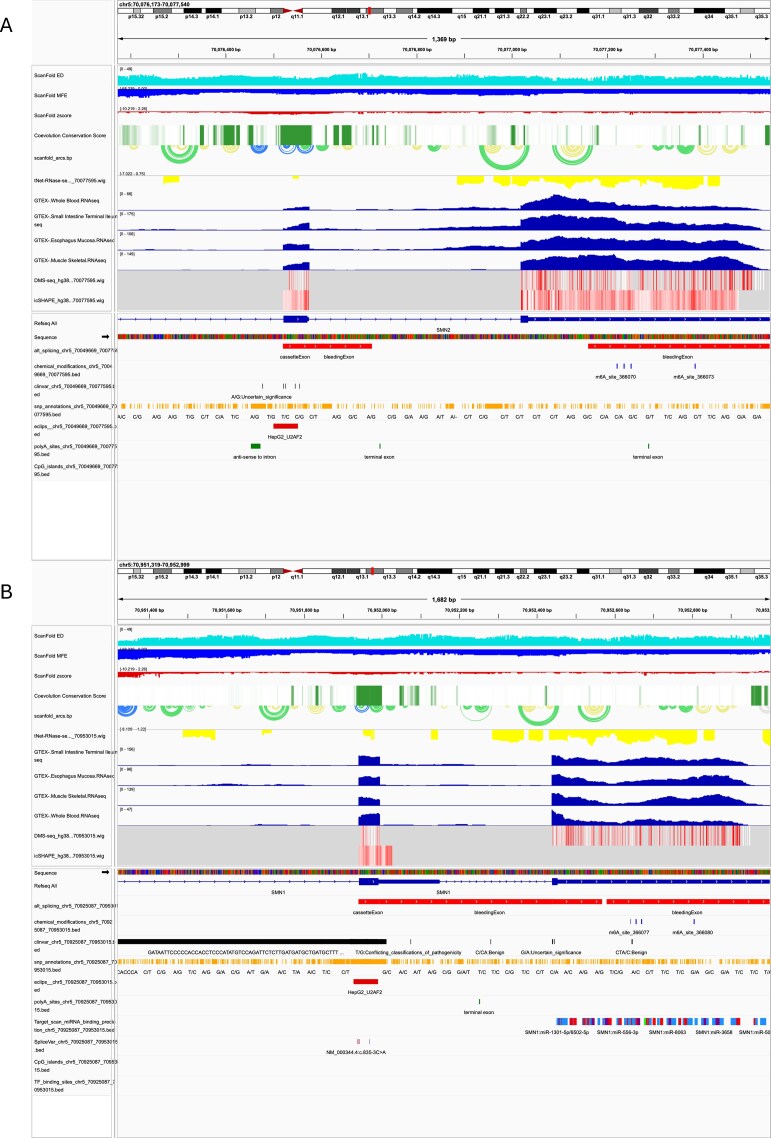
Integrated annotation view of the SMN2 and SMN1 loci reveals a disease-causing structural switch. Multi-track visualization of the human (**A**) *SMN2* and (**B**) *SMN1* gene loci generated by the RNA-Annotator pipeline. Each panel displays an identical set of curated annotation tracks, allowing for a direct comparison between the two paralogs. From top to bottom, tracks include: ScanFold-predicted metrics (Ensemble Diversity, MFE, and *z*-score), evolutionary conservation (phastCons), ScanFold-predicted base-pairing arcs, enzymatic probing (tNET-RNase-seq), tissue-specific expression from GTEx (whole blood, small intestine, esophagus, and skeletal muscle), *in vivo* chemical probing (DMS-seq and icSHAPE), and a variety of functional annotations including alternative splicing, chemical modifications, ClinVar variants, CpG islands, eCLIP protein-binding sites, SNPs, and miRNA-binding sites. While the genes show broad similarities in expression and conservation, a distinct structural difference is visible in the ScanFold arc plot over exon 7, which is denser and contains more significant low *z*-score pairings (blue arcs) in the *SMN2* locus compared to *SMN1*.

As a use case to demonstrate the synergistic power of the RNAStructuromeDB and RNA-Annotator, we analyzed the clinically relevant paralogs SMN1 and SMN2. These genes are nearly identical, yet a single C-to-T transition in exon 7 of SMN2 disrupts a critical splicing enhancer, leading to exon skipping that causes spinal muscular atrophy [56]. For each gene, the pipeline generates not only a full suite of IGV-ready visualization tracks but also a corresponding set of data-rich .tsv and .csv files containing detailed information for every annotation (Supplementary Folders S1 and S2). This dual output allows for both high-level visual inspection and deep quantitative analysis. By applying RNA-Annotator to both genes, we generated a multi-layered view that integrates structural predictions with a rich set of functional annotations, enabling a comprehensive, comparative analysis from a single command (Fig. 4A and B).

The power of this approach lies in its ability to rapidly collate disparate data types into a single, coherent view. For instance, while it is impractical to display all tissue expression data simultaneously, a representative subset of GTEx tracks (e.g. small intestine, esophagus mucosa, and skeletal muscle) reveals that both SMN1 and SMN2 are broadly expressed, indicating that their differential splicing, not their expression level, is the key driver of the disease phenotype [57]. Similarly, the sequence similarity between the two paralogs is reflected in the consistent prediction of miRNA-binding sites downstream of exon 7 and evolutionary conservation, as measured by phastCons scores, is also high across exon 7 in both genes, underscoring its functional importance. Furthermore, enzymatic probing data from tNET-RNase-seq shows a largely similar cleavage pattern, suggesting that the overall accessibility to ribonucleases is comparable.

**Figure 5.**
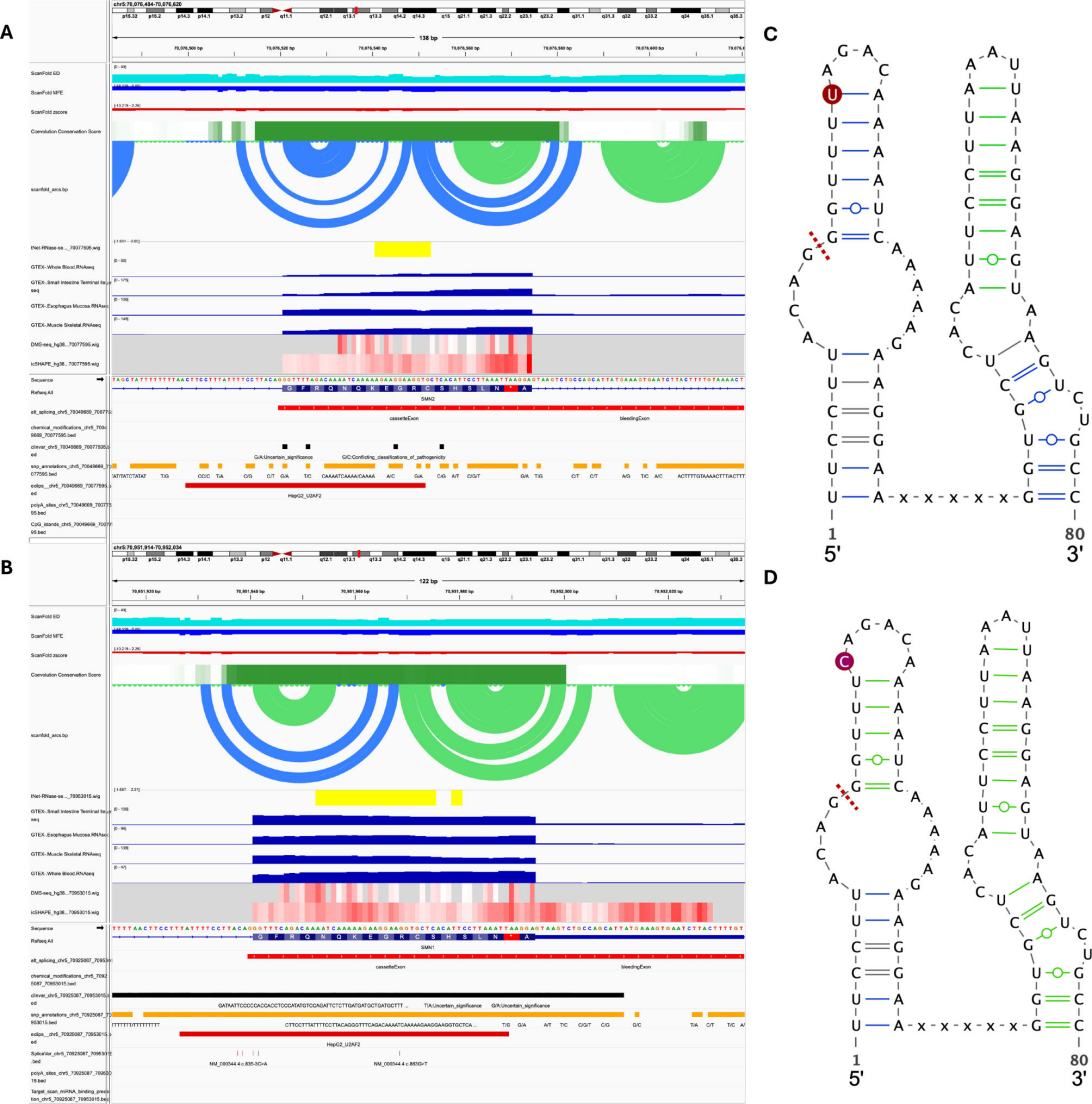
High-resolution view reveals the structural and functional consequences of the pathogenic variant in *SMN2* Exon 7. A detailed comparison of the local structural and functional landscape surrounding exon 7 in (**A**) *SMN2* and (**B**) *SMN1*. The IGV panels show a dramatic shift in the ScanFold-predicted secondary structure, with the *SMN2* locus exhibiting a dense cluster of low *z*-score base pairs (blue arcs) that are absent in *SMN1*. This region of increased stability in *SMN2* coincides with the binding site for the splicing factor U2AF2, which is visible in the eCLIP track for *SMN1*. Panels (**C**) and (**D**) show 2D structural models of the consensus ScanFold structures for *SMN2* and *SMN1*, respectively, visualized with VARNA. The models explicitly show the formation of a more stable and extended hairpin in *SMN2* (C) compared to the more modest structure in *SMN1* (D). The positions of the key C-to-U variant (in RNA) are highlighted in red, demonstrating the structural basis for the sequestration of the exonic splicing enhancer and subsequent exon 7 skipping in SMN2 Base pairs are colored grey for MFE pairs, green for *z*-scores between −1 and −2, and blue for *z*-scores ≤ −2). Red dashed lines indicate the splice sites; sequestration in panel (C) promotes exon 7 skipping, while accessibility in panel (D) permits exon inclusion.

Despite these extensive similarities, the RNA-Annotator output immediately highlights the critical differences stemming from the single nucleotide variant. The ScanFold arc plot for SMN2 displays a visibly different and denser pattern of arcs over exon 7, colored in blue to reflect more significant z-scores (Fig. 5). This indicates the formation of a more stable and ordered local secondary structure, which is the direct cause of the reported “cassetteExon” alternative splicing event also annotated in the view. This rapid, holistic visualization demonstrates the pipeline’s ability to move from a high-level comparative overview to pinpointing a specific, disease-causing structural switch, providing a powerful tool for hypothesis generation and data interpretation.

A high-resolution view of exon 7 and its flanking introns reveals the direct structural consequences of the C-to-T transition in SMN2 (Fig. 5). While the overall sequence and conservation profile (phastCons) remain nearly identical between SMN1 and SMN2, the ScanFold predictions show a dramatic shift in the thermodynamic stability of the local RNA secondary structure. In the wild-type SMN1 gene, exon 7 is part of a relatively modest structure, characterized primarily by green arcs (*z*-score between −1 and −2) (Fig. 5B). In stark contrast, the pathogenic variant in SMN2 induces the formation of a significantly more stable and ordered stem-loop structure, visualized by a dense cluster of blue arcs (*z*-score ≤ −2) spanning the 5′ splice site region of exon 7 (Fig. 5A). This computational prediction is further corroborated by *in vivo* chemical probing data. Specifically, the DMS reactivity profile shows a marked decrease in reactivity for several Adenosine residues within the newly formed hairpin in SMN2, indicating they have become protected and inaccessible within a double-stranded stem, perfectly reflecting the predicted structural change.

These findings align with prior enzymatic and chemical probing studies showing that the C6U mutation stabilizes the terminal stem-loop 1 (TSL1) at the 5′ end of exon 7—extending its stem to sequester the 3′ splice site and exonic splicing enhancers—while also enhancing TSL2 stability at the 3′ end to hide the 5′ splice site, as visualized in our extended hairpin models (Fig. 5C and D) [58–61]. This structural rearrangement has direct functional implications for splicing factor recruitment. The eCLIP track shows a distinct binding peak for the splicing factor U2AF2 within the exon 7 region of SMN1. In SMN2, this binding site is predicted to be sequestered within the more stable stem-loop, likely disrupting its recognition. To visualize this structural switch more explicitly, we modeled the consensus structures using VARNA (Fig. 5C and D). These models confirm that the SMN2 variant creates a more stable, extended hairpin, providing a clear structural basis for the sequestration of a known exonic splicing enhancer (ESE) and the subsequent exclusion of exon 7 from the mature mRNA.

## Discussion

In this study, we present the RNAStructuromeDB, the first comprehensive, computational map of RNA secondary structures across the entire human pre-mRNA transcriptome. By applying the ScanFold pipeline to over 230 000 transcripts, we have generated a massive dataset that provides a penetrating view into the structural landscape of human RNA. This resource is complemented by two powerful toolkits: the RNA-Annotator pipeline for integrating structure with rich functional context, and an analysis suite for the in-depth characterization of identified structural motifs. Crucially, these components form a complete ecosystem that moves beyond a static data repository, enabling a dynamic workflow from transcriptome-wide exploration to the detailed analysis of individual RNA targets.We observed a striking enrichment of complex structures like multibranch loops in 5′UTRs—a finding consistent with their known roles in translational regulation—while 3′UTRs and introns emerged as hotspots for structural diversity. Furthermore, our analysis uncovered clear evidence of local stability compensation, a key biophysical principle where RNA structures maintain thermodynamic stability through local sequence adjustments. For example, we demonstrated an inverse relationship between hairpin stem length and GC content, and a positive correlation between apical loop size and the GC content of the supporting stem. Our large-scale computational approach independently rediscovers these known biophysical rules not only validates the methodology but also provides a quantitative, transcriptome-wide confirmation of these principles, offering a rich dataset for future studies on the evolution of RNA structure.

The true power of this work lies in the synergy between the RNAStructuromeDB and the RNA-Annotator pipeline. As our *SMN1/SMN2* case study demonstrates, this integrated approach transforms the immense challenge of target validation into a streamlined and powerful process. In a matter of minutes, a user can collate dozens of disparate data types—from clinical variants and protein binding sites to chemical probing data and evolutionary conservation—onto a single, coherent structural canvas. This ability to rapidly overlay functional context onto a structural framework is critical for prioritizing therapeutic targets. It allows researchers to quickly filter thousands of potential RNA targets down to a manageable few that possess desirable characteristics, such as containing a stable, functionally important, and accessible structural motif. This pipeline therefore represents a significant step forward in accelerating the discovery pipeline for both RNA-targeted therapeutics and functional genomics.

In practice, RNA-Annotator serves as the programmatic interface (“API”) to this workflow, enabling reproducible, command-line retrieval of ScanFold outputs together with integrated annotation layers as IGV-ready tracks and detailed tabular results. Importantly, RNA-Annotator is not designed to independently validate predictions or to automatically “call” druggable sites; rather, it provides the structured evidence needed for target nomination and prioritization, which remains context- and modality-dependent (e.g. ASO versus small molecule).

Although all computational RNA structures are predictions and may differ from in-cell conformations, we emphasize that the primary utility of RNAStructuromeDB is not “a single definitive structure,” but a genome-scale structural baseline paired with rich functional context. Experimental probing datasets (e.g. DMS/SHAPE collections such as RASP) are invaluable, but they are inherently condition-dependent (cell type, tissue, reagent, and protocol) and remain sparse or absent for large fractions of the pre-mRNA transcriptome—especially introns. Our resource complements these efforts by providing transcriptome-wide ScanFold maps and an integrated RNA-Annotator layer that overlays ∼20 annotation tracks (e.g. RBP binding, expression, splicing, variants, conservation, and available probing) so users can rapidly identify where predicted structure aligns with functional signals. In this way, RNAStructuromeDB supports target nomination and hypothesis generation, and helps prioritize a small set of high-value motifs for downstream experimental validation under the relevant biological conditions.

We also recognize that comparative sequence analysis—specifically the detection of co-varying base pairs—remains the gold standard for structural validation [62]. Covariation analysis provides powerful evolutionary evidence for base pairing and, particularly when combined with chemical probing data such as SHAPE, can yield structural models of high accuracy [63]. However, implementing high-quality covariation analysis at the transcriptome scale presents significant challenges, as reliable detection requires deep sequence alignments that are often difficult to generate automatically, particularly for rapidly evolving or repetitive intronic regions. Furthermore, recent statistical frameworks have highlighted the risk of false positives arising from phylogenetic artifacts in automated analyses [64]. Because of that we employed the ScanFold approach, which evaluates thermodynamic stability using single-sequence shuffling and does not rely on multiple sequence alignments. This strategy ensures feasibility and consistency across the entire transcriptome. To complement this, RNA-Annotator integrates vertebrate conservation scores (phastCons) and diverse functional annotation layers (e.g. RBP occupancy, expression, variants, splicing, and probing). This combination enables robust, genome-scale target nomination and prioritization even when covariation is not feasible, while still providing critical conservation information to support downstream covariation analysis when appropriate.

While the primary focus of this study was to generate and curate a comprehensive, transcriptome-wide dataset of predicted RNA secondary structures, this resource establishes a foundation for many deeper analyses. Future work could explore motif distributions across different *z-*score thresholds, compare structural trends between introns and exons, or evaluate distinctions between pre-mRNA and mature transcripts. Additional analyses could also examine correlations between low-*z*-score regions and intronic transposons or investigate RBP-binding preferences relative to local substructures such as loop size or branch complexity. These directions extend beyond the current scope but represent logical next steps to extract further biological insight from the RNAStructuromeDB. In future releases, we also plan to extend this framework to additional species, while maintaining the current focus on the human pre-mRNA transcriptome as the most direct foundation for RNA-targeted therapeutic discovery

Looking forward, the vast and multi-layered dataset we have generated provides an ideal foundation for developing next-generation predictive models. A traditional, rule-based approach to identifying “druggable” RNA sites is inherently limited. As the *SMN1/SMN2* example vividly illustrates, the biology of RNA is exquisitely complex; a single nucleotide change can induce profound structural and functional consequences that would be difficult to predict with a simple set of rules. The sheer number of variables and their non-linear interactions make this problem intractable for classical methods. This is precisely the kind of challenge where large language models (LLMs) and deep learning excel. We envision a future in which all the features generated by ScanFold and collated by RNA-Annotator for millions of structural motifs can be used to train a sophisticated RNA language model [65–68]. Such a model would have the potential to learn the deep, intricate patterns of sequence, structure, and function, ultimately allowing it to predict, with much higher accuracy, which sites are not just structured, but truly functional and therapeutically tractable.

Finally, we acknowledge the limitations of our study. The ScanFold pipeline, in its current implementation, cannot process transcripts longer than 200 000 nucleotides, which excludes a small number of the largest human genes. More significantly, the consensus-building algorithm in ScanFold-Fold resolves competition for pairing partners by dropping interactions, which can occasionally result in an open parenthesis in the final dot-bracket notation that is never closed. While this affects a very small fraction of the data, it represents a known issue where the model’s simplification may not perfectly reflect the biological reality of competing conformations. Additionally, the current version of ScanFold does not predict pseudoknots or G-quadruplex (G4) structures, both of which can play important roles in RNA folding and function. Incorporating these complex structural motifs represents another important area for future improvement. Future work will aim to address these computational limitations and further refine the structural models.

In conclusion, the RNAStructuromeDB and its associated toolkits provide a foundational resource that we believe will have a broad and lasting impact on the RNA research community. By making this dataset fully publicly accessible, we hope to empower new discoveries in basic RNA biology, accelerate the development of novel RNA therapeutics, and provide the training data for the next generation of predictive biological models.

## Supplementary Material

lqag044_Supplemental_File

## Data Availability

All data are available through the RNAStructuromeDB or the Supplementary Information: Supplementary Files S7 and S8, containing large dictionaries of RNA structures, are available on Zenodo (https://doi.org/10.5281/zenodo.17807277). The complete, final curated collection of all datasets used in RNA Annotator is available for download as a single compressed folder (resources_data_sets) from Zenodo (https://doi.org/10.5281/zenodo.16953760). The RNA-Annotator source code, Conda environment file (for one-step installation), and user manual are available at https://github.com/moss-lab/RNA-Annotator-v1 and https://doi.org/10.5281/zenodo.19210233. Example outputs for the SMN1/SMN2 case study are provided in the repository https://github.com/moss-lab/RNA-Annotator-v1/tree/main/examples and as a Supplementary Folder. The ScanFold2.0 source code is available at https://github.com/moss-lab/ScanFold2.0 and https://doi.org/10.5281/zenodo.19210246. The custom Python pipeline used for global structural feature analysis and LOGO-based sequence conservation analyses is available at (https://github.com/jacopomanigrasso/RNA_2D_stats/ and https://doi.org/10.5281/zenodo.19218217).
