# Supplementary material for: RNAStructuromeDB: a transcriptome-wide database of predicted RNA secondary structures with integrated APIs for functional annotation and RNA-targeted drug discovery
Source: NAR Genom Bioinform. 2026 Apr 30;8(2):lqag044. doi: 10.1093/nargab/lqag044 (PMC13129543; doi:10.1093/nargab/lqag044)
Supplement: lqag044_Supplemental_File [file lqag044_supplemental_file.pdf]

Supplementary Figures

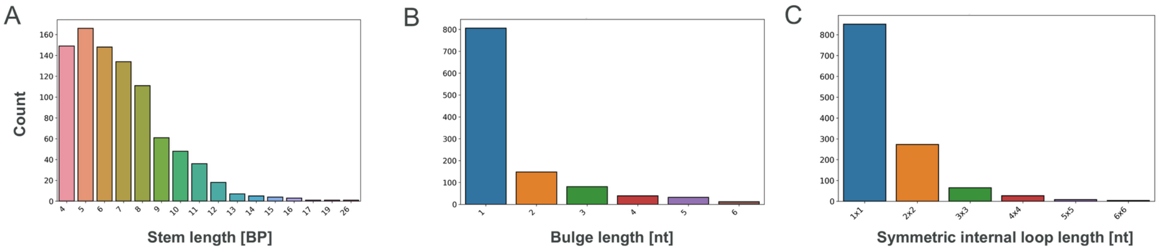

Supplementary Figure S1. Structural features of stems closing multibranch loops. (A) Distribution of fully base-paired stems. (B-C) Distribution of bulges and symmetric internal loops found in non-fully paired stems.

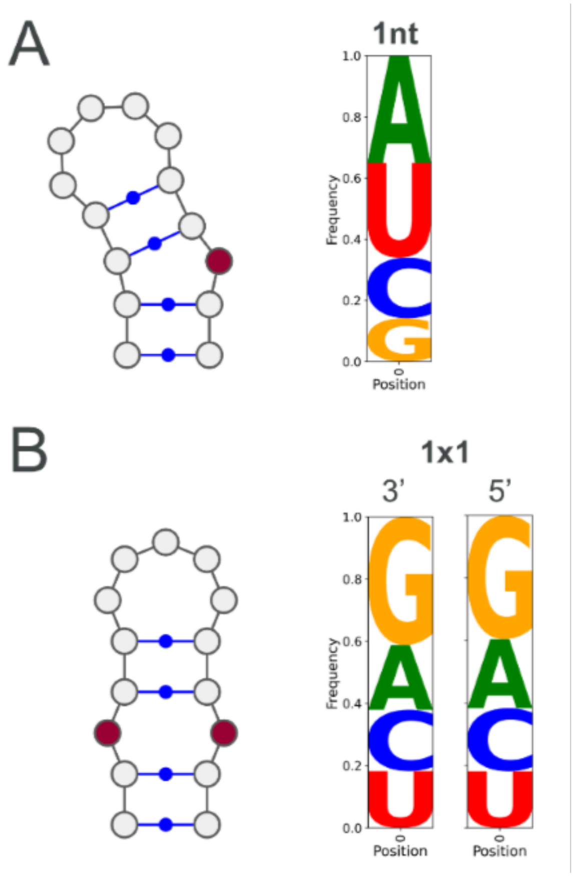

Supplementary Figure S2. LOGO analysis of bulges and symmetric internal loops found in stems closing multibranch loops. (A) Sequence composition of single-nucleotide bulges showing predominance of adenine and uracil residues. (B) Sequence analysis of 1×1 symmetric internal loops demonstrating guanosine enrichment up to 40%.

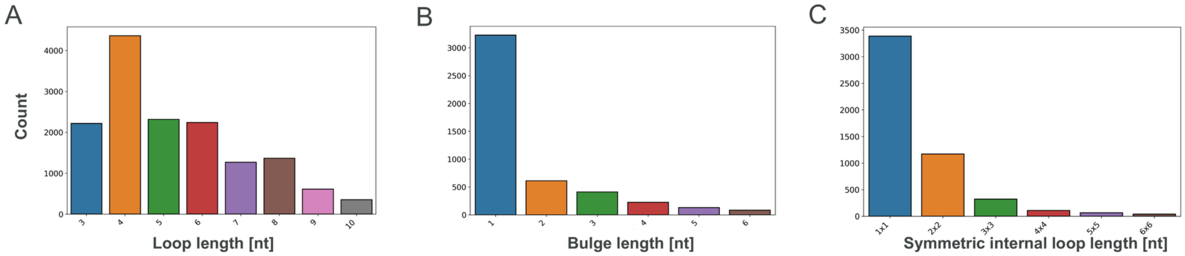

Supplementary Figure S3. Structural features of hairpin stems. (A) Distribution of fully base-paired hairpin stems. (B-C) Distribution of bulges and symmetric internal loops found in non-fully paired hairpin stems.

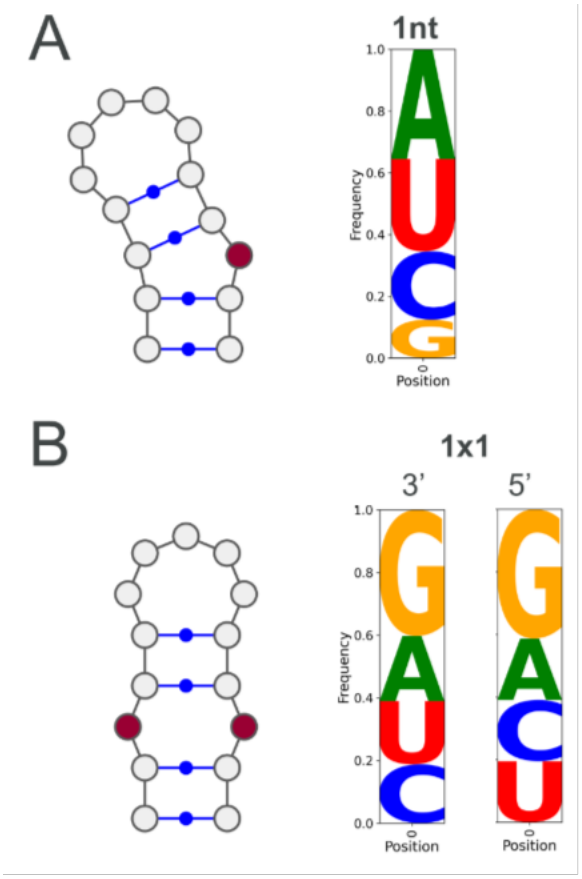

Supplementary Figure S4. LOGO analysis of (A) bulges and (B) symmetric internal loops found in hairpin stems.

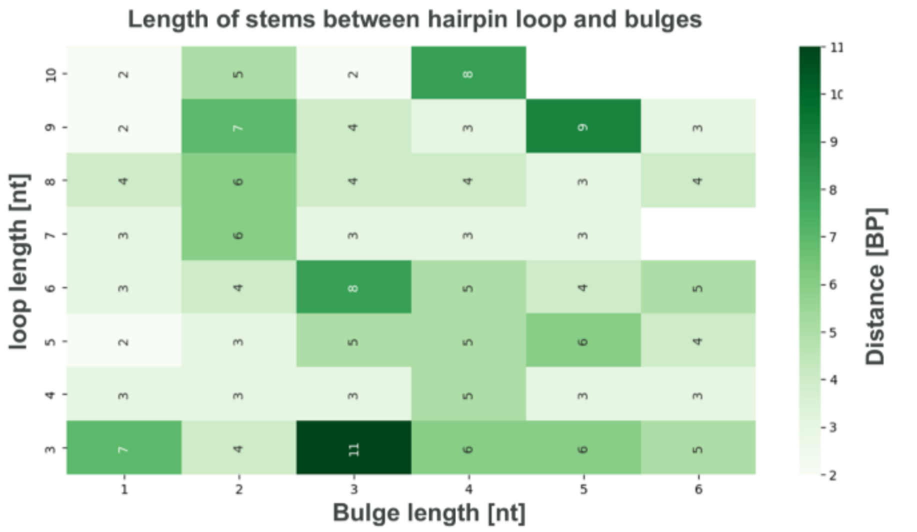

Supplementary Figure S5. Spatial organization of bulges in hairpins. Distance analysis showing the positioning of bulges relative to apical loops, with 1-nucleotide bulges typically located 2-3 base pairs from apical loops, except for triloops which show

7 base pair separation. Heat map displays bulge length versus apical loop length with distance color coding.

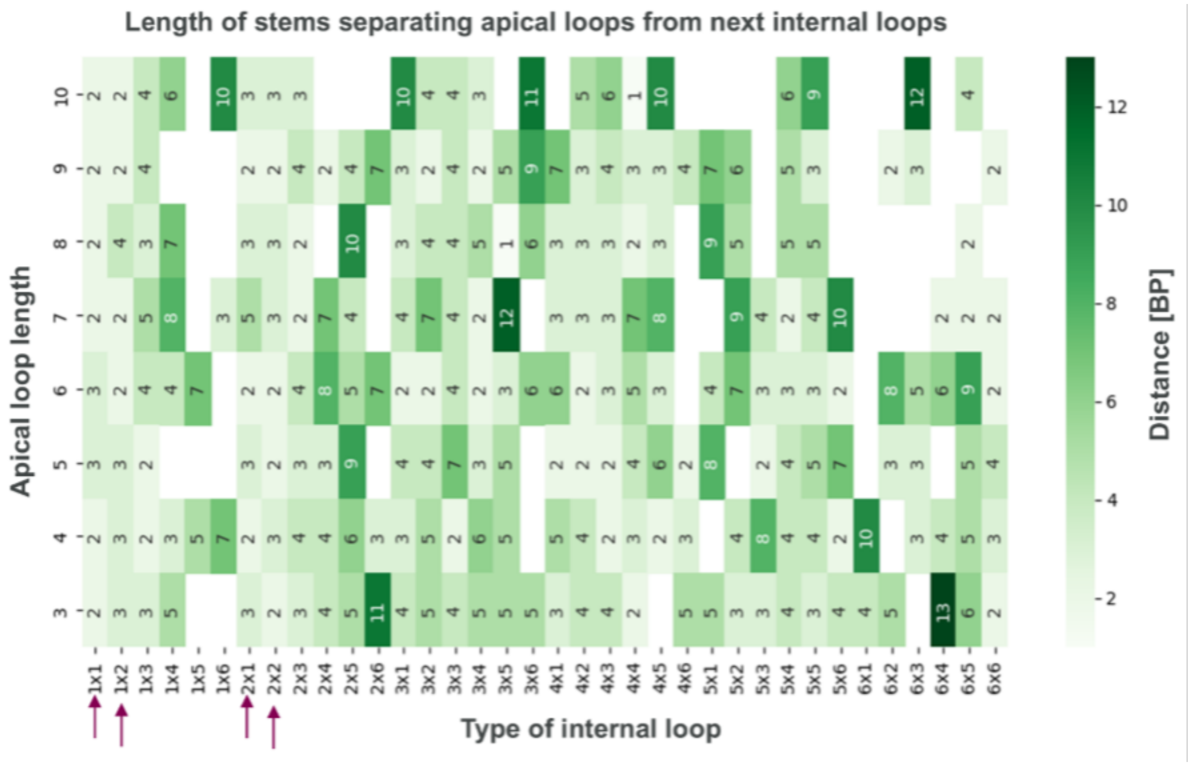

Supplementary Figure S6. Spatial organization of internal loops in hairpins. Distance distribution analysis for symmetric and asymmetric internal loops showing preferential positioning 2-3 base pairs from apical loops. Heat map displays internal loop type and length versus apical loop length with distance color coding.
